# Demethylation of Cancer/Testis Antigens and CpG ODN Stimulation Enhance Dendritic Cell and Cytotoxic T Lymphocyte Function in a Mouse Mammary Model

**DOI:** 10.1155/2013/196894

**Published:** 2013-11-04

**Authors:** Jun-Zhong Sun, Lei Gao, Li Gao, Wei Wang, Nan Du, Juan Yang, Ling Wan, Fang Liu, Li-li Wang, Li Yu

**Affiliations:** ^1^Department of Hematology, Chinese PLA General Hospital, Beijing 100853, China; ^2^Department of Oncology, The First Affiliated Hospital of Chinese PLA General Hospital, Beijing 100048, China; ^3^Department of Pharmacy, The First Affiliated Hospital of Chinese PLA General Hospital, Beijing 100048, China; ^4^Department of Hematology, China-Japan Friendship Hospital, Beijing 100029, China; ^5^No. 535 Hospital of Chinese People's Liberation Army, Huaihua, Hunan 418000, China

## Abstract

*Background*. Cancer/testis antigens (CTAs) are ideal targets for cancer immunotherapy in virtue of their restricted expression profile in normal tissues. However, CTA-targeted immunotherapy has been rather disappointing clinical setting for CTAs are downregulated by cytosine-phosphate-guanosine (CpG) methylation in their promoter regions, so that tumor cells have low immunogenicity. *Methods*. We reinduced mouse CTA P1A through demethylation process and generated P1A-specific cytotoxic lymphocytes (CTLs) by immunizing BALB/c (H-2^d^) mice with dendritic cells pulsed with a P1A-specific peptide and CpG oligodeoxynucleotide (ODN) immune adjuvant. *Results*. We found that demethylation and CpG ODN immune adjuvant stimulation facilitated DC maturation and enhanced the allogenic capacity of P1A-specific CTLs against target cells both *in vitro* and *in vivo*. *Conclusions*. Our results suggested that CTA induction and immune adjuvant stimulation is a feasible strategy in cancer immunotherapy.

## 1. Background

Immunotherapy has long been considered as a potent anticancer therapy due to its powerful tumor inhibition [1]. Although immunotherapy was indeed able to induce cytotoxic lymphocytes (CTLs) that recognize tumor antigens *in vitro*, it did not show equally potent antitumor activity *in vivo*, due to the lack of either tumor cell immunogenicity [2] or tumor-associated antigens for specific recognition by immune cells [3]. Therefore, major challenges, including activating antitumor immune cells and enhancing tumor cell immunogenicity to facilitate effective recognition, occurred at present. 

CpG oligodeoxynucleotide (ODN), extensively investigated as an immune adjuvant [4], is recognized by toll-like receptor 9 (TLR9) and induces immune responses, during which effective presentation of tumor-specific antigen (TSA) to T lymphocytes is essential for subsequent activation of CTLs that ultimately eliminate tumor cells. However, tumor-specific CTLs do not work well due to the lack of tumor cell immunogenicity. 

Cancer/testis antigens (CTAs) are considered a promising class of tumor antigens for therapeutic cancer vaccines on account of their restricted expression profiles in normal tissues and also due to the testis being an immunoprivileged site [5]. Recently, adoptive T-cell therapy has achieved prospective clinical results, including cancer regression in metastatic melanoma patients [6]. However, most of the initial cancer immunotherapies against CTAs resulted in poor clinical outcome owing to immune tolerance [7], with one main reason that CTA hypermethylation in the process of cancer transformation [8], that might downregulate or silence gene expression in cancer cells, makes the recognition of immunotherapeutic vaccines or cancer-specific CTLs difficult.

In consequence, we hypothesized that reinduction of CTAs by demethylation and concomitant stimulation by CpG ODN could induce an effective immune response against cancers expressing the related CTAs. In this study we would reinduce mouse CTA P1A with 5-aza-2-deoxycytidine (5-aza) and employed P1A peptide and immune adjuvant CpG ODN to stimulate DC maturation and the proliferative, as well as cytotoxic activities of T lymphocytes for the purpose of developing a new useful procedure for immunotherapy.

## 2. Methods

### 2.1. Cell Lines and Cell Culture

A20 (mouse B-lymphoid tumor), CT26 (mouse colon adenocarcinoma), and 4T1 (mouse mammary carcinoma) cell lines were purchased from Cell Culture Center, Chinese Academy of Medical Science. Cells were cultured in RPMI 1640 supplemented with 10% fetal bovine serum (FBS) plus 1% penicillin/streptomycin and 1% glutamine. Cultures were maintained in a 5% CO_2_-humidified incubator at 37°C, and experiments were performed with cells in the exponential growth phase.

### 2.2. Chemicals, Drugs, and Reagents

Antibodies for cell phenotype identification or apoptosis analysis: Monoclonal antibodies (mAb) against CD90, CD4, CD8, CD80, CD86, CD11c, mouse immunoglobulin isotype controls, MACS CD4 microbeads, and MACS CD90.1 microbeads were purchased from BD (Franklin Lakes, NJ, USA). Murine GM-CSF, interleukin-4 (IL-4), and mouse CFSE were from Dojindo Co. (Kumamoto, Japan). The CpG-containing phosphorothioated oligodeoxynucleotides (CpG ODN Class-B 1826: 5′TCC ATG GAC GTT CCT GAG CGT T3′) and H-2L^d^-restricted synthetic peptide derived from P1A (P1A 35−43: LPYLGWLVF) were synthesized by Beijing Saibaisheng Biological Engineering Technology Co. (Beijing, China) based on the previous reports [9, 10].

### 2.3. Treatment of Tumor Cells with 5-Aza *In Vitro*

A20, CT26, and 4T1 cells were treated with 5-aza with a final concentration of 3 *μ*mol/L and cultured for 48 hours as described above. Control cultures were grown under similar experimental conditions but without 5-aza.

### 2.4. DNA Isolation and Methylation-Specific PCR

Genomic DNA was isolated with a Qiagen Dneasy kit (Hilden, Germany) according to the manufacturer's instructions, and bisulfite-modified genomic DNA was amplified as reported previously [11]. The following PCR primers were used: methylated forward, 5′-TTA  AGT  GCG  TTA  TTA  CGT  TTG  GTT  TTT  AC-3′ and reverse, 5′-ATA  ACC  GAT  TAT  TTA  ATA  CAA  AAA  TCG  ACG-3′, unmethylated forward, 5′-GAT  TAA  GTG  TGT  TAT  TAT  GTT  TGG  TTT  TTA  T-3′ and reverse, 5′-ACA  TAA  CCA  ATT  ATT  TAA  TAC  AAA  AAT  CAA  CA-3′. 

### 2.5. RNA Extraction and Reverse Transcription PCR (RT-PCR)

Total RNA was extracted with the Invitrogen SuperScript RNA extraction kit (Carlsbad, CA, USA). The sequences of primers for P1A and glyceraldehyde-3-phosphate (GAPDH) were as follows: P1A, forward: 5′-CGG  AAT  TCT  GTG  CCA  TGT  CTG  ATA  ACA  AGA  AA-3′ and reverse: 5′-CGT  CTA  GAT  TGC  AAC  TGC  ATG  CCT  AAG  GTG  AG-3′; GAPDH, forward: 5′-GCC  TCG  TCC  CGT  AGA  CAA  AA-3′ and reverse: 5′-CCA  TTC  TCG  GCC  TTG  ACT  GT-3′.

### 2.6. DC Maturation by CpG ODN and P1A-Specific Peptide Pulsing

Bone marrow (BM) monocytes isolated by Ficoll density gradient centrifugation were then cultured in RPMI 1640. On day 3, GM-CSF (20 ng/mL) and murine IL-4 (10 ng/mL) were added. After 7 days' culture, CpG ODN, P1A peptide, or CpG ODN (10 *μ*g/mL) + P1A peptide (10 *μ*g/mL) were added and further cultured for 3 days. The cells and culture supernatant were separately collected for further cell phenotype and cytokine level assays. Supernatant IL-6 and TNF-*α* levels were assayed using a mouse IL-6 enzyme-linked immunosorbent assay (ELISA) kit and a mouse TNF-*α* ELISA kit (Diaclone, Besançon, France) according to the manufacturer's instructions. 

### 2.7. Generation and Activation of P1A CTL

The stimulation effects of DCs on T cell proliferation and cytotoxic activity were determined using a previously described method [12]. T lymphocytes were separated by T-cell separation columns and used as response cells. The DCs were exposed to CpG ODN and P1A-specific peptide for 3 days and used as stimulating cells. The response cells and stimulating cells were cocultured for 96 hours at a ratio of 20 : 1. The cells and the culture supernatant were then separately collected for further experiments cytokine level assays. Granzyme B and perforin levels in the supernatants were measured using the mouse granzyme and mouse perforin ELISA kits (Diaclone). 

### 2.8. Cytotoxic Activity of P1A CTL: Apoptosis Assay

To measure the cytotoxic activity of P1A-specific CTLs on 4T1 cells, we detected apoptosis using the Annexin V method. Briefly, demethylated 4T1 cells (10^6^) were cocultured with P1A-specific CTLs at indicated effector-to-target (E/T) ratios for 16 hours and then stained with Annexin V and 7-AAD for detection by flow cytometry.

### 2.9. *In Vivo* Experiments of P1A CTL

All mouse experiments were performed with female BALB/C purchased from the Institute of Laboratory Animal Science, Chinese Academy of Medical Science. The experimental protocols were approved by the Chinese Laboratory Animal Center. All experiments were carried out according to the standards of animal care as outlined in the Guide for the Care and Use of Experimental Animals of Peking Union Medical College. Briefly, the mice were maintained and handled under aseptic conditions with 12/12-hour light-dark conditions and had free access to food and water during the study. 

The *in vivo* antitumor effect was evaluated with xenograft-transplanted BALB/C mice according to the previously reports [13]. 4T1 cells were transplanted into the flank of mice. On day 6, the mice bearing 4T1 cells were randomly divided into four groups (*n* = 6 per group), and 5-aza (1.0 mg/kg) was injected intraperitoneally twice a day for 5 day. On day 13, 5 × 10^5^ specially treated CTLs were injected into the tail vein. Tumor volumes were measured every other day during the whole experiment period and calculated using the reported equation [14]. On day 21, the mice were euthanized, and the tumors were weighed for further analysis. Tumor inhibition rate was calculated as inhibition rate = (*a* − *b*)/*a* × 100%, where “*a*” and “*b*” represent the average weights of the control and treatment groups, respectively.

### 2.10. Statistical Analysis

All data from *in vitro* and *in vivo* experiments were analyzed by Pearson *χ* ^2^ tests or Student's *t*-tests, and values of *P* < 0.05 were considered statistically significant.

## 3. Results

### 3.1. DNA Methylation and Gene Expression of CTA P1A in Cancer Cell Lines

**Figure 1:**
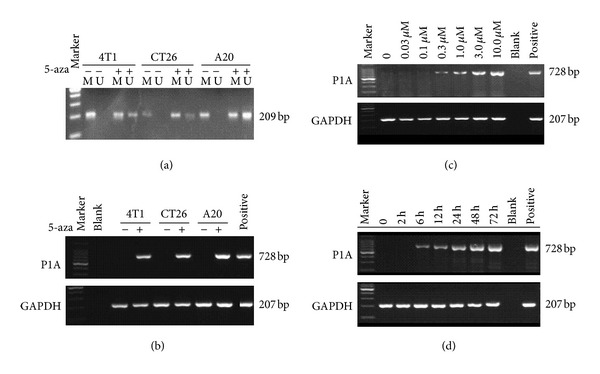
Methylation status of the P1A gene promoter and dose- and time-dependent P1A induction with 5-aza in 4T1 cells. 4T1, CT26, and A20 cell lines were treated with or without 3 *μ*M 5-aza for 48 h, and (a) methylated levels were shown, −, not treated with 5-aza and +, 5-aza treated cells (M, methylated DNA; U, unmethylated DNA). (b) P1A mRNA levels detected by RT-PCR. (c) 5-aza dose response. 4T1 cells were treated for 48 h with 5-aza at concentrations of 0, 0.03, 0.1, 0.3, 1.0, 3, or 10 *μ*M. Blank, negative control and P, positive control. (d) Time course 5-aza treatment at a fixed concentration of 3.0 *μ*M. 4T1 cells were incubated with 5-aza for 0, 2, 6, 12, 24, 48, or 72 h. The P1A expression was detected with P1A-specific primers. GAPDH was amplified as a control to demonstrate cDNA integrity. The positive lane contained EL9611 tumor cells that express high level of P1A.

We firstly detected the DNA methylation status of P1A gene in A20, CT26, and 4T1 murine cells using a methylation-specific PCR. After 5-aza treatment, both methylated and unmethylated products were shown in Figure 1(a). We further measured P1A expression levels by RT-PCR and analyzed whether 5-aza-induced P1A mRNA expression was dose- and time-dependent. As shown in Figures 1(b), 1(c), and 1(d), P1A mRNA was undetectable without previous 5-aza, while after exposure to 5-aza, P1A mRNA was induced in 4T1 cells with dose- and time-dependent pattern. EL9611, a murine erythroblastic leukemia cell line expressing a high level of P1A [15], was used as positive control. In our studies, 5-azacytidine exposure for 48 h at different concentrations is not cytotoxic to the cells (data not shown).

We also investigated the relationship between the duration of 5-aza exposure and P1A gene expression. We found that a 6-hour 5-aza exposure with a final concentration of 3.0 *μ*M was sufficient to induce P1A expression; longer exposure time could increase CTA expression until 48 hour (Figure 1(d)). These results demonstrate that 5-aza can induce P1A expression in a dose- and time-dependent manner in 4T1 cells.

### 3.2. Effect of P1A-Specific Peptide and CpG ODN on DC Phenotype and Function

**Figure 2:**
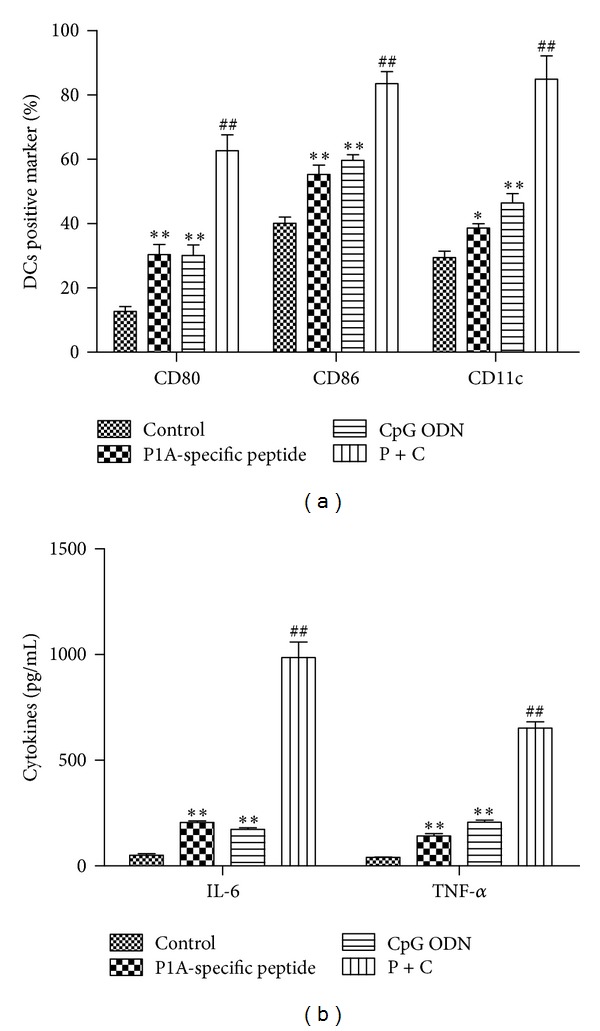
The effect of P1A-specific peptide and CpG ODN on DC maturation. (a) DCs positive for CD80, CD86, and CD11c by flow cytometry. (b) ELISA measurements of IL-6 and TNF-*α* release by cultured DCs. IL-4 and GM-CSF, P1A-specific peptide, CpG ODN, or P1A-specific peptide + CpG ODN were added to DCs for 96 hour. Values are the means ± SD (*n* = 3) of three individual experiments. *P < 0.05 and **P < 0.01 versus control; ^#^ *P* < 0.05 and ^##^ *P* < 0.01 versus group treated with P1A-specific peptide or CpG ODN.

We assessed the phenotypes of DCs pulsed with IL-4 coupled with GM-CSF, P1A-specific peptide, CpG ODN, or P1A-specific peptide + CpG ODN. As shown in Figure 2(a), the number of DCs positive for CD80, CD86, and CD11c after induction with P1A peptide + CpG ODN were 64.35 ± 8.2%, 85.41 ± 10.5%, and 83.61 ± 6.4%, respectively, which were significantly higher than those in DCs treated with either P1A-specific or CpG ODN alone (*P* < 0.05) or IL-4 and GM-CSF (*P* < 0.01). When mouse BM cells were further cultured in the presence of P1A-specific peptide and CpG ODN for 3 days, IL-6 and TNF-*α* (both markers associated with mature DCs) were secreted into the culture media. As shown in Figure 2(b), IL-6 and TNF-*α* levels were significantly higher in P1A-specific peptide + CpG ODN cells than those in either the P1A-specific peptide or the CpG ODN alone groups (*P* < 0.01), suggesting that P1A-specific peptide and CpG ODN stimulated DC maturation.

### 3.3. Effect of CpG ODN- and P1A-Specific Peptide-Treated DCs on Allogenic T Lymphocyte Proliferation and Cytotoxic Activity

**Figure 3:**
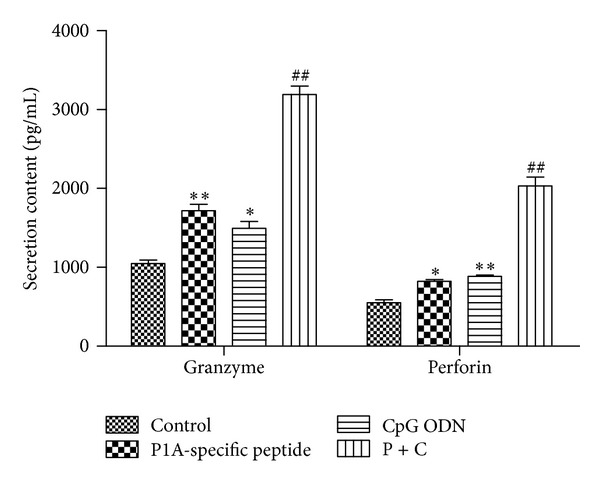
Granzyme B and perforin release from CTLs cocultured with DCs treated using different protocols (IL-2, P1A-specific peptide, CpG ODN, or P1A-specific peptide + CpG ODN). Both granzyme B and perforin secretion were markedly increased upon treatment with P1A-specific + CpG ODN (*P* < 0.01). Values are the means ± SD (*n* = 3) of three individual experiments. *P < 0.05 and **P < 0.01 versus control; ^#^ *P* < 0.05 and ^##^ *P* < 0.01 versus group treated with P1A-specific peptide or CpG ODN.

We cocultured lymphocytes isolated from H-2L^d^-restricted BALB/C mouse spleen with DCs at 20:1 for 96 hours and measured granzyme B and perforin secretion by DC-stimulated CTLs (Figure 3). Both were markedly increased upon treatment with P1A-specific peptide + CpG ODN, illustrating that P1A-specific peptide and CpG ODN enhanced CTL cytotoxicity through increase of granzyme B and perforin release.

### 3.4. *In Vitro* Cytotoxic Activity of P1A-Specific CTL on H-2L^d^-Restricted 4T1 Cells

**Figure 4:**
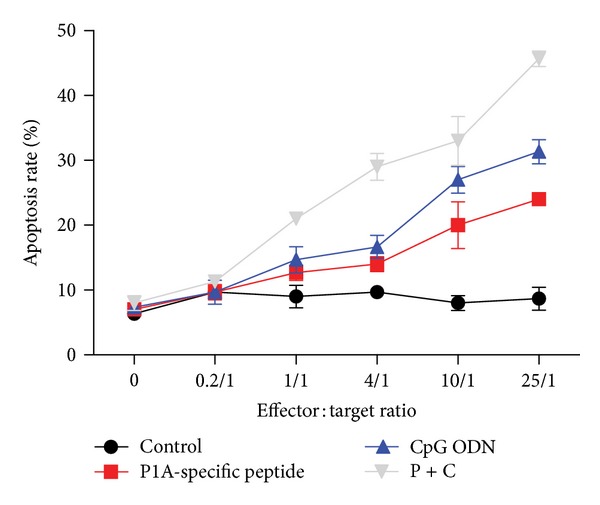
*In vitro* cytotoxic activity of P1A-specific CTLs on H-2L^d^-restricted 4T1 cells. 4T1 tumor cells were treated with 5-aza for 24 h and then cultured with P1A-specific CTLs at different effector/target ratios for 16 h. The number of apoptotic 4T1 cells (Annexin V positive/7-AAD (early apoptosis) cells) increased in a dose-dependent manner after incubation with P1A-specific peptide + CpG ODN-treated P1A-specific CTLs. The differences were significant (*P* < 0.01) compared to the P1A-specific peptide or CpG ODN alone. CTLs did not cause similar apoptosis in control group, even if at a higher effect/target ratio of 25 : 1.

To determine the activity of P1A-specific CTLs against P1A-expression tumor cells, we cultured target cells (4T1) treated with 5-aza coupled with specially treated CTLs at different effector/target ratios for 16 hour. As shown in Figure 4, the proportion of apoptotic 4T1 cells was significantly higher in the P1A-specific peptide + CpG ODN group than that in the P1A-specific peptide or CpG ODN alone or control groups (IL-4 and GM-CSF, *P* < 0.05). Nearly all the tumor cells died when the effect/target ratio reached 25 : 1. These data demonstrate that the cytotoxic effect of P1A-specific CTLs was enhanced by P1A-specific peptide and CpG ODN stimulation.

### 3.5. Antitumor Effect of H-2L^d^-Restricted P1A-Specific CTLs *In Vivo*

**Figure 5:**
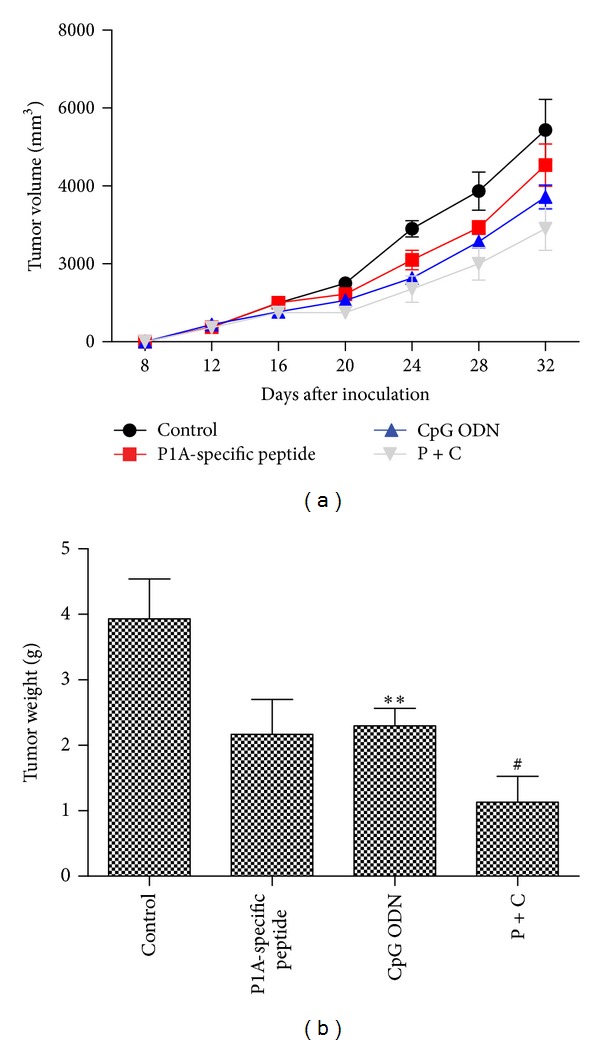
Antitumor Effect of H-2L^d^-restricted P1A-specific CTLs *in vivo*. We established an mouse model bearing 4T1 cells. (a) H-2L^d^-restricted P1A-specific CTLs significantly inhibited 4T1 cell growth in BALB/C mice treated with P1A-specific peptide + CpG ODN. (b) Tumor weight in the P1A-specific peptide + CpG ODN treated group was significantly lower than that of the control groups. *P < 0.05, **P < 0.01 versus Control; ^#^ *P* < 0.05, ^##^ *P* < 0.01 versus group treated with P1A-specific peptide or CpG ODN.

We then studied the effect of specially treated CTLs on tumor growth using a BALB/C tumor-bearing mouse model. Briefly, 2 × 10^3^ 4T1 cells were transplanted into mice on day 1. Five days later, 5-aza and specially treated CTLs were administered as mentioned above. As shown in Figure 5, P1A-specific peptide and CpG ODN-stimulated CTLs significantly inhibited the growth of 4T1 cells in 5-aza-treated BALB/C mice. Compared with the control group (not treated with P1A or CpG ODN), after CTL administration, the tumor volume was barely increased compared with pre-treatment (Figure 5(a)). Tumor weight in P1A-specific peptide and CpG ODN-stimulated CTLs group was significantly lower than that of the control groups (Figure 5(b)). The inhibitory rate of H-2L^d^-restricted P1A-specific CTLs on tumor growth reached a level of 79.8%, whereas the inhibitory rate of the control groups was less than 35.5%.

## 4. Discussion

Some tumors allow them to escape from immune surveillance and destruction, that have puzzled the oncologist for a long time [16]. Hence, counteracting tumor escape mechanism is a key issue for successful immunotherapy [17]. Although CTAs are considered to be ideal targets for cancer immunotherapy, their expression levels are often decreased or silenced by DNA methylation in the promoter region of CT genes [18], that make it difficult for antigen-specific CTLs to recognize and kill tumor cells. Recent studies have shown that demethylating agents modulating CTA expression could be a useful strategy helpful for cancer immunotherapy. High CTA expression levels facilitate tumor cell or tissue to be recognized by the body's immune system and ultimately lead to tumor clearance by CTLs. In our study, the P1A gene expression was regained with 5-aza in murine tumor cell lines normally without P1A expression, suggesting that the low/no expression of the P1A gene was due to hypermethylation in the CpG region, that was the key mechanism for immunologic escape and tolerance. 

However, several current studies reported that cancer vaccines have little efficacy if tumor-specific cytotoxins are not incorporated with immune helpers, such as immune adjuvant or helper peptides [19]. When using single epitope wild-type p53, peptide-specific CD8+ T cells were generated in just one-third of healthy donors or subjects with cancer. In this study, we induced DC maturation with a P1A-specific peptide and CpG ODN and found that mature DC phenotypes were upregulated and cytokine release was significantly enhanced. These results suggest that reinduction of the P1A gene incorporated with immune adjuvant CpG ODN may be a feasible and useful cell-based immunotherapy strategy.

TLR agonists have been widely used in cancer therapy due to their inducement to potent antitumor immune responses [20]. Binding of CpG ODN and TLR9 facilitated their ability to induce DC maturation and promoted the differentiation of T helper (Th0) into Th1 [21–23]. Effective CTL activation is the key step in cancer immunotherapy. Mature DCs provide an interface between the innate and adaptive immune systems, acting as antigen-presenting cells [23], and may enhance cytotoxic T lymphocyte activation and function. In this study, we cocultured mouse T lymphocytes with P1A-specific peptide and CpG ODN-stimulated DCs and found that the function of P1A-specific CTLs was enhanced. Additional experiments showed that P1A-specific CTLs possessed antitumor activity against 4T1 tumor cells both *in vitro* and *in vivo*, providing strong evidence that reinduction of P1A CTA and CpG ODN stimulation can be used to enhance the efficacy of cancer immune therapy.

## 5. Conclusions

We observed that demethylation and CpG ODNs immune adjuvant stimulation facilitated DC maturation and enhanced the capacity of allogenic P1A-specific CTLs against target cells both *in vitro* and *in vivo*. And our results suggest that induced CTA and immune adjuvant stimulation is a feasible strategy for adoptive immunotherapy for cancer.
